# Effectiveness of Non-Immersive Virtual Reality on Gross Motor Function, Balance, and Functional Independence in Children with Cerebral Palsy: A Systematic Review with Meta-Analysis

**DOI:** 10.3390/jcm14217582

**Published:** 2025-10-25

**Authors:** Joaquín Perez-Carcamo, Jordan Hernandez-Martinez, Edgar Vásquez-Carrasco, Diego Fernandez-Cardenas, Braulio Henrique Magnani Branco, Cristian Sandoval, Eduardo Carmine-Peña, Francisca Peña, Juan Aristegui-Mondaca, Pablo Valdés-Badilla

**Affiliations:** 1 G-IDyAF Research Group, Department of Physical Activity Sciences, Universidad de Los Lagos, Osorno 5290000, Chile; joaquinalejandro.perez@alumnos.ulagos.cl; 2 Department of Physical Activity Sciences, Universidad de Los Lagos, Osorno 5290000, Chile; jordan.hernandez@ulagos.cl; 3 Department of Education, Faculty of Humanities, Universidad de la Serena, La Serena 1700000, Chile; 4 School of Occupational Therapy, Faculty of Psychology, Universidad de Talca, Talca 3465548, Chile; edgar.vasquez@utalca.cl; 5 Centro de Investigación en Ciencias Cognitivas, Faculty of Psychology, Universidad de Talca, Talca 3465548, Chile; 6 School of Occupational Therapy, Health Sciences Faculty, Catholic University of Maule, Talca 3530000, Chile; dfernandezc@ucm.cl; 7 Graduate Program in Health Promotion, Cesumar University (UniCesumar), Maringá 87050-900, Brazil; braulio.branco@unicesumar.edu.br; 8 Escuela de Tecnología Médica, Facultad de Salud, Universidad Santo Tomás, Los Carreras 753, Osorno 5310431, Chile; cristian.sandoval@ufrontera.cl; 9 Departamento de Medicina Interna, Facultad de Medicina, Universidad de La Frontera, Temuco 4811230, Chile; 10 Carrera de Medicina, Facultad de Medicina, Universidad de La Frontera, Temuco 4811230, Chile; e.carmine01@ufromail.cl; 11 Department of Physical Activity Sciences, Faculty of Education Sciences, Universidad Católica del Maule, Talca 3530000, Chile; juan.aristegui@alu.ucm.cl; 12 Sports Coach Career, Faculty of Life Sciences, Universidad de Viña del Mar, Viña del Mar 2520000, Chile

**Keywords:** activities of daily living, non-immersive virtual reality (VR), motor skills disorders, neuronal Plasticity, rehabilitation, children, postural balance

## Abstract

**Background/Objectives:** This systematic review with meta-analysis synthesizes current evidence on the effectiveness of non-immersive virtual reality (VR) interventions in enhancing gross motor function, balance, and functional independence in children with cerebral palsy (CP). **Methods:** A systematic search was performed across six databases (PubMed, Web of Science, Scopus, MEDLINE, CINAHL Complete, and Psychology and Behavioral Sciences Collection) to identify randomized controlled trials (RCTs) published up to July 2025. Primary outcomes included gross motor function (GMFM-D/E), balance (Pediatric Balance Scale, PBS), and functional independence (WeeFIM). Risk of bias was assessed using the RoB 2 tool, and the certainty of evidence was evaluated with GRADE. **Results:** From 1233 retrieved records, 13 RCTs involving 624 participants fulfilled the inclusion criteria. Pooled analyses demonstrated significant improvements with non-immersive VR in gross motor function (GMFM-D: ES = 2.04, *p* = 0.02; GMFM-E: ES = 2.02, *p* < 0.001), balance (PBS: ES = 1.34, *p* = 0.02), and functional independence (WeeFIM: ES = 0.99, *p* < 0.001). **Conclusions:** Non-immersive VR interventions were associated with meaningful gains in gross motor function, balance, and independence in children with CP. Significant differences were consistently observed in GMFM-D, GMFM-E, PBS, and WeeFIM outcomes when compared with control groups.

## 1. Introduction

Cerebral palsy (CP) encompasses a group of neurological disorders affecting movement and posture, resulting from non-progressive injury to the developing brain during the prenatal, perinatal, or early postnatal stages [1]. Although the underlying brain lesion does not progress, the clinical manifestations often emerge as impairments in motor control, posture, and gait throughout childhood [1,2]. CP is among the most prevalent neurodevelopmental conditions in childhood [3] and is frequently associated not only with motor deficits but also with cognitive, sensory, and perceptual difficulties. Due to these motor challenges, children with CP often struggle with routine physical activities that are essential for maintaining independence and quality of life [4,5]. When left untreated or insufficiently managed, these limitations can contribute to additional long-term health complications [6,7,8]. While conventional physical therapy is effective, it is not always engaging for children, particularly those with difficulties in attention and motivation [9]. In this context, virtual reality (VR) has gained increasing attention as an interactive and enjoyable therapeutic tool that can enhance participation and adherence to rehabilitation programs [10].

Interactive video games delivered through virtual reality (VR) can provide an engaging and motivating environment that facilitates motor learning and improves adherence to therapy in children with CP [11]. VR technologies are generally categorized into immersive, semi-immersive, and non-immersive formats. Immersive VR employs head-mounted displays together with inertial motion-tracking sensors to capture users’ orientation and movements, allowing them to interact within a three-dimensional virtual space characterized by a strong sense of presence and sensory immersion [12]. In contrast, non-immersive VR uses conventional screens and motion sensors, enabling users to interact with virtual environments while remaining anchored to the physical world [13]. Among these modalities, immersive and non-immersive VR are the most frequently used in pediatric neurorehabilitation. This approach often incorporates commercial gaming platforms, such as the Nintendo Wii, Xbox Kinect, or PlayStation Move, with interactions displayed on standard monitors or televisions. By combining multisensory feedback with game-based features, these systems enhance motivation, support motor adaptation, and foster neuroplastic changes in children with neurological disorders [14,15,16].

A VR-based program conducted by Fidan and Genç [17] using the Xbox Kinect reported significant improvements in gross motor function, balance, and postural control among children with spastic CP (*p* = 0.000). In a similar vein, Tarakci et al. [18] found that non-immersive VR physiotherapy with the Nintendo Wii led to significant gains in balance and functional independence (*p* < 0.05). Likewise, Mouhamed et al. [19] demonstrated that training with the Wii balance board produced greater improvements in balance in children with ataxic CP compared with conventional physical therapy, as measured by the Pediatric Balance Scale (PBS, *p* = 0.001). Collectively, these findings support the growing body of evidence suggesting that VR-based interventions enhance motor performance and learning in CP by engaging mechanisms that facilitate the completion of functional tasks [20].

Despite promising findings, diversity in study design, frequency, duration, and intervention technology has prevented the generalization of these results. This meta-analysis attempts to summarize the current evidence for the effectiveness of non-immersive VR-based interventions on gross motor function, balance, and functional independence in children with CP. In addition, subgroups were analyzed to explore how key aspects of the intervention, especially training dose, might influence the efficacy of non-immersive VR therapies.

## 2. Materials and Methods

### 2.1. Protocol and Registration

This systematic review was conducted in line with PRISMA recommendations [21], and the protocol was prospectively registered in PROSPERO (CRD420251040489).

### 2.2. Eligibility Criteria

To ensure greater homogeneity in neurodevelopmental stage, this review focused on randomized controlled trials (RCTs) published up to July 2025 that examined non-immersive VR interventions in children with CP under the age of 12 years. No language restrictions were applied. Eligible studies were required to follow the PICOS framework (Table 1) and include both baseline and post-intervention assessments of at least one of the following outcomes: gross motor function (GMFM-D/E), balance (PBS), or functional independence (WeeFIM). Comparisons were accepted against either active or inactive control groups. Exclusion criteria were limited to studies that did not meet these parameters, such as nonrandomized designs, review articles, study protocols, and those using immersive or semi-immersive VR systems. In addition, single clinical case reports of participants over 18 years with CP were excluded, as they do not provide experimental control or comparative data.

### 2.3. Information Search Process and Databases

Six databases were searched: PubMed and MEDLINE, Scopus, Web of Science (Core Collection), CINAHL Complete, and Psychology and Behavioral Sciences Collection. Intermittent random searches were performed from December 2024 to July 2025. This period corresponds to the final operational phase of screening and validation; however, all studies published up to July 2025 were considered regardless of publication year, as described in the PROSPERO registration (CRD420251040489). The search strategy developed was purposely designed to include a full range of search terms pertaining to non-immersive VR interventions in childhood CP, with a particular focus on gross motor function, balance, and functional independence. The search string used was (“virtual reality”[MeSH Terms] OR “virtual reality” OR “nonimmersive virtual reality” OR “immersive virtual reality” OR “VR-based therapy” OR “virtual reality therapy” OR “virtual reality rehabilitation”) AND (“cerebral palsy”[MeSH Terms] OR “cerebral palsy” OR “CP”)AND (“children”[MeSH Terms] OR “child”[MeSH Terms] OR “pediatric patients” OR “children” OR “pediatrics” OR “adolescents”) AND (“gross motor function”[MeSH Terms] OR “gross motor function” OR “motor skills” OR “motor performance” OR “rehabilitation”[MeSH Terms] OR “rehabilitation” OR “gait” OR “balance” OR “mobility” OR “upper limb function” OR “upper extremity function” OR “coordination” OR “postural control”). Two independent reviewers also consulted with two experts in the field to abstract inclusion and exclusion criteria and identify potential studies. The following experts are included in this list: (i) possess a PhD degree in sports and/or rehabilitation sciences and (ii) have peer-reviewed publications on physical performance in pediatric and/or clinical populations that are indexed in journals with impact factors such as the Journal Citation Reports^®^. Additionally, all papers included in the best estimate of the database were screened again by 30 July 2025, for any retractions or errata.

### 2.4. Data Collection and Selection Procedure

References were input into EndNote X9 (Clarivate Analytics, Philadelphia, PA, USA). Two independent examiners (J.P.-C. and J.H.-M.) conducted independent searches, screened titles and abstracts, removed duplicates, and evaluated full-text articles for eligibility. The level of agreement between reviewers was calculated using Cohen’s Kappa coefficient and the percentage of agreement, yielding Kappa = 0.87 and 94.3% agreement, indicating almost perfect consistency [23]. No discrepancies between evaluators have been reported to date. Furthermore, the reference listings of the included studies and expert recommendations were manually reviewed to identify additional pertinent publications. All completely reviewed articles were documented with specific reasons for exclusion in accordance with predefined eligibility criteria.

### 2.5. Methodological Quality Assessment

The methodological quality of the included trials was evaluated using the TESTEX scale, a tool specifically developed for studies involving exercise-based interventions [24]. In this review. The instrument assigns a maximum of 15 points, comprising 5 points for study design and methodological quality, and 10 points for reporting quality, as described by Smart et al. [24]. Although low scores were regarded as a possible reason for exclusion, none of the included studies were removed on this basis.

### 2.6. Data Synthesis

The following variables were extracted and analyzed from each of the selected studies: (i) first author and year of publication; (ii) country of origin; (iii) study design; (iv) baseline clinical characteristics of participants; (v) sample size in both experimental and control group; (vi) mean age of participants; (vii) type of activities performed in non-immersive VR intervention and the control condition; (viii) training load, including intervention duration, session frequency, and session length; (ix) exercise intensity when reported; (x) primary outcome measures, including gross motor function (e.g., GMFM-D, GMFM-E), balance (e.g., PBS), and functional independence (e.g., WeeFIM), when available; and (xi) main findings and reported effects.

### 2.7. Risk of Bias in Individual Studies

The risk of bias for each included study was independently assessed by two reviewers (J.P.-C. and J.H.-M.) using the RoB 2 tool [25]. A third reviewer (E.V.-C.) verified the classifications across the following domains: randomization process, adherence to the intended intervention, completeness of follow-up data, measurement of outcomes, and selective reporting of results. Each domain was rated as “low risk,” “some concerns,” or “high risk”.

### 2.8. Summary Measures for Meta-Analysis

This review included a meta-analysis component, with full protocol details available in the PROSPERO registry (CRD420251040489). Meta-analyses were conducted only when at least three studies reported on the same outcome variable [26]. For each eligible trial, effect sizes (ES; Hedges’ g) were calculated using pre- and post-intervention means and standard deviations. Primary outcomes were gross motor function (GMFM-D, GMFM-E), balance (Pediatric Balance Scale), and functional independence (WeeFIM). Whenever possible, standardized change scores were applied to ensure comparability of effect estimates across studies [26].

Analyses were performed using Comprehensive Meta-Analysis 3.0 software, with statistical significance set at *p* ≤ 0.05 [27]. Between-study heterogeneity was examined with Cochrane’s Q test [28] and the I^2^ statistic, where thresholds of >25%, 25–50%, and >50% indicated low, moderate, and high inconsistency, respectively.

A random-effects model was applied, and independent analyses of specific components were performed to explore potential sources of heterogeneity that could influence the effects of the interventions.

### 2.9. Factor Analysis of Single-Training

Single-factor analyses were conducted for moderators such as participants’ age, intervention duration (weeks), and training frequency (sessions per week) [29]. When appropriate, subgroup and single-factor analysis were performed using the median split method [30]. Median values for each moderator were calculated only when at least two studies provided relevant data [31]. This procedure minimized bias when multiple groups from the same study shared identical moderator values.

When trials included two experimental groups, only the group corresponding to the pre-established comparator relevant to the outcome of interest was included to prevent overrepresentation. To further reduce heterogeneity, median values were derived specifically from the subset of studies contributing data to the outcome of interest, rather than applying a single global median across all included studies.

### 2.10. Assessment of the Certainty of the Evidence

The certainty of evidence was assessed using the GRADE (Grading of Recommendations, Assessment, Development, and Evaluation) framework [32,33]. Evidence quality was categorized as very low, low, moderate, or high. Since all included studies were RCTs, the initial rating for each outcome began as high certainty. Downgrading was applied when concerns were identified regarding risk of bias, consistency, coherence, accuracy, precision, transparency of reporting, or potential publication bias. Two authors (J.P.-C. and J.H.-M.) independently performed the GRADE assessments.

## 3. Results

### 3.1. Study Selection

**Figure 1:**
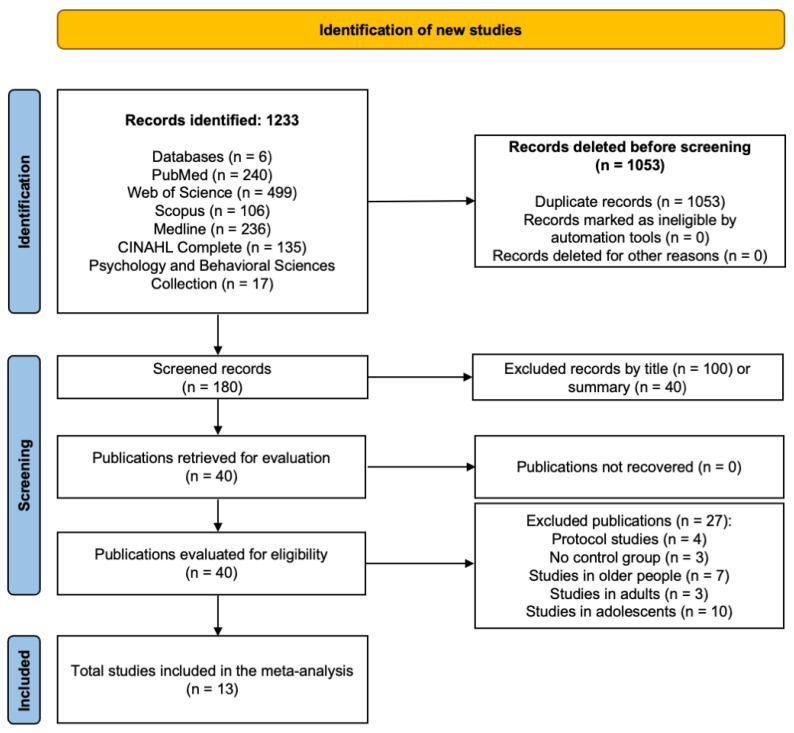
PRISMA flowchart.

The search process is summarized in Figure 1. A total of 1233 records were initially identified. After removing 1053 duplicates, 180 unique records remained for screening based on title, abstract, and keywords. Of these, 100 were excluded by title and 40 by abstract for not meeting the eligibility criteria. The remaining 40 full-text articles were retrieved for detailed assessment.

Following full-text evaluation, 20 studies were excluded due to age-related mismatches (seven involving older adults, three involving adults, and 10 involving adolescents), four were protocol papers, and three lacked a control group. Ultimately, 13 randomized controlled trials met the inclusion criteria and were incorporated into the meta-analysis [9,17,18,19,34,35,36,37,38,39,40,41,42].

### 3.2. Methodological Quality

The 13 included studies were assessed using the TESTEX scale. All studies achieved at least 53% of the maximum score, reflecting moderate to high methodological quality.

Specifically, two studies scored 8/15 [9,17], six scored 9/15 [18,35,36,37,38,39], three scored 11/15 [19,40,41], one scored 11/15 [42], and one achieved 12/15 [34]. These results suggest that, overall, the included trials demonstrated adequate experimental design, appropriate participant follow-up, and clear outcome reporting. Since none of the studies were rated as having low methodological quality, no exclusions were made on this basis (Table 2).

### 3.3. Characteristics of the Studies

All the selected RCTs used non-immersive VR aimed at improving gross motor function, balance, or independence in children with CP. Interventions were classified into five categories according to the type of VR system used: (i) interactive sessions with Nintendo Wii and/or Xbox Kinect [9,17,19,34,35,39,40]; (ii) stationary cycling workouts [38]; (iii) treadmill training [35]; (iv) tailor-made programs for interactive therapy applications without commercial consoles but still within non-immersive frameworks [41,42]; and (v) hybrid protocols, where non-immersive VR was combined with additional technologies [36]. The programs lasted between 2 and 12 weeks, with a frequency of 2–5 days depending on the week and consultation intervals ranging from 20–135 min. Most interventions were conducted in clinical settings, although some were conducted at home. With respect to geographical distribution, studies have been conducted in various countries: South Korea [35], Brazil [36], India [9,34], the United Kingdom [37], China [39], Taiwan [38,41], Egypt [19], Lebanon [42], and Turkey [17,18,39]. All 13 selected studies were RCTs [9,17,18,19,34,35,36,37,38,39,40,41,42]. The characteristics of the participants and interventions across studies are detailed in Table 3.

### 3.4. Risk of Bias

**Figure 2:**
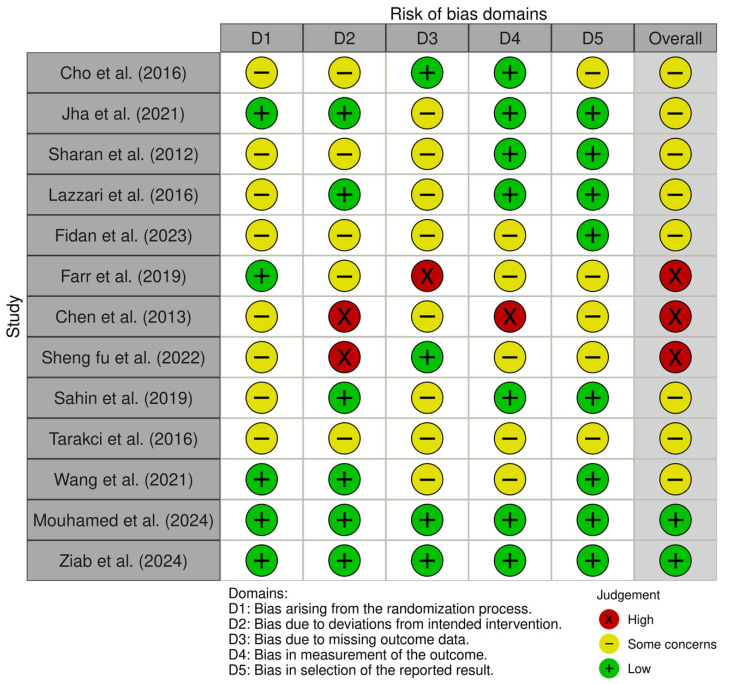
Risk of bias tools: traffic lights chart. The studies included are: Sharan et al. [9], Fidan and Genç [17], Tarakci et al. [18], Mouhamed et al. [19], Jha et al. [34], Cho et al. [35], Lazzari et al. [36], Farr et al. [37], Chen et al. [38], Fu et al. [39], Şahin et al. [40], Wang et al. [41], and Ziab et al. [42].

**Figure 3:**
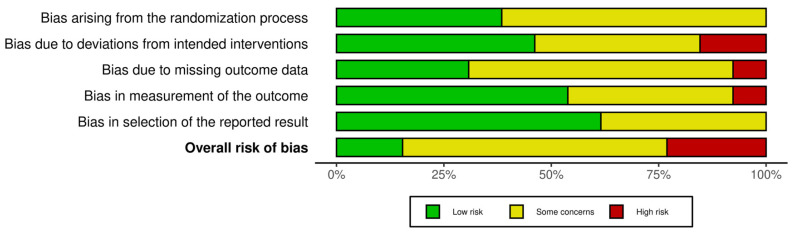
Risk of bias tools: Summary chart by domain.

Overall, the included studies demonstrated acceptable methodological quality, with most rated as having either a low risk of bias or some concerns. Of the 13 studies assessed, three were classified as high risk [36,37,38], primarily due to deviations from the intended intervention (D2) and incomplete outcome data (D3). Eight studies [9,17,18,34,35,36,40,41] were judged as presenting some concerns, indicating moderate methodological risk, but not to an extent that would compromise their overall validity. Only two studies [19,42] were rated as low risk across all domains, which increases confidence in their findings. A summary of risk-of-bias assessments is presented in Figure 2 and Figure 3.

### 3.5. Sample Characteristics

Across the 13 included studies, sample sizes ranged from 15 to 62 participants [9,17,18,19,34,35,36,37,38,39,40,41,42], yielding a total of 437 children diagnosed with CP. The overall mean age was 8.7 years. Most participants were classified within levels I to III of the Gross Motor Function Classification System (GMFCS) [17,18,38,40,41,42].

Among the studies that reported sex distribution (n = 202 participants), 65% were male and 35% female, although proportions varied across individual trials. Interventions were predominantly delivered in clinical or school-based environments, and all participants underwent non-immersive VR protocols targeting improvements in gross motor function, balance, or functional independence.

### 3.6. Dosing and Conducted Interventions

Interventions lasted between 2 and 12 weeks, with regular training 2 to 5 days per week and sessions lasting between 20 and 135 min [9,17,18,19,34,35,36,37,38,39,40,41,42]. Four studies did not report the intensity of physical activity [9,36,37,38]. Furthermore, no studies have assessed intensity via CERT (Children’s Evaluation of Exertion Chart). The type of video game console used varied across the studies analyzed. The Xbox Kinect console was used in four studies [17,18,19,40]. Six studies used Nintendo Wii and Wii Fit [9,18,19,37,39,40], whereas three studies did not specify the type of console used in the sessions [34,36,41]. Four types of non-immersive VR interventions were used: (i) interactive sessions with Nintendo Wii and/or Xbox Kinect [9,17,19,34,37,39,40]; (ii) stationary cycling training [38]; (iii) treadmill training [34]; (iv) tailored programs for interactive therapy applications, without commercial consoles but still within non-immersive frameworks [41,42]; and (v) hybrid protocols combining non-immersive VR with other technologies [36].

### 3.7. Meta-Analysis Results

Meta-analyses demonstrated effects in favor of non-immersive VR for: GMFM-D (Hedges’ g = 2.04; 95% CI: 0.73–3.35; *p* = 0.02; I^2^ = 89.9%), GMFM-E (Hedges’ g = 2.02; 95% CI: 0.97–3.08; *p* < 0.001; I^2^ = 84.7%), PBS (Hedges’ g = 1.34; 95% CI: 0.17–2.52; *p* = 0.02; I^2^ = 93.4%), and WeeFIM (Hedges’ g = 0.99; 95% CI: 0.65–1.33; *p* < 0.001; I^2^ = 32.2%). The specific meta-analyses for GMFM-D, GMFM-E, PBS, and WeeFIM can be observed in the Supplementary Material (Figures S1–S7). A summary of the main findings is shown in Table 4.

### 3.8. Moderator Analysis

Moderator analyses were performed when at least two studies provided data for a specific training-related variable. Four subgroup analyses were conducted for the PBS, examining session duration (minutes), weekly training frequency, and total number of sessions. These analyses were intended to determine whether specific intervention characteristics influenced the effectiveness of non-immersive VR on balance outcomes in children with CP.

### 3.9. Certainty of Evidence

For gross motor function, the risk of bias was deemed not serious, with no concerns related to inconsistency, indirectness, or imprecision. Accordingly, the certainty of evidence was rated as high, reflecting strong confidence in the results.

In contrast, the evidence for balance was rated as moderate to high certainty, downgraded due to serious concerns about risk of bias. Similarly, functional independence, evaluated across four studies, also showed serious risk-of-bias concerns, resulting in a moderate to high certainty rating. In both cases, no additional issues were identified in the domains of inconsistency, indirectness, or imprecision, and no other modifying factors affected the assessment.

These findings indicate that while the evidence supporting improvements in gross motor function is robust, conclusions regarding balance and functional independence should be interpreted with caution due to methodological limitations (Table 5).

### 3.10. Adverse Events and Adherence

Across the 13 included RCTs, no serious adverse events related to non-immersive VR interventions were reported. Minor issues such as mild fatigue or temporary lapses in attention were occasionally observed but were not systematically recorded and were not considered clinically significant.

Adherence was generally high, with most studies reporting session attendance rates of ≥85%, reflecting good compliance. However, only five studies explicitly documented adherence or dropout rates [19,34,40,41,42]. Reported dropout rates were low, and when reasons for withdrawal were provided, they were unrelated to the intervention (e.g., scheduling conflicts).

These findings suggest that non-immersive VR interventions are safe, well-tolerated, and feasible for children with CP in both clinical and home settings. The playful and interactive nature of the sessions likely contributed to strong motivation and adherence, reinforcing the potential of VR-based rehabilitation as a promising tool in pediatric care.

## 4. Discussion

### 4.1. Gross Motor Function

The GMFM-D (ES = 2.04) and GMFM-E (ES = 2.02) tests conducted in the present meta-analysis demonstrated substantial enhancements in gross motor function in favor of non-immersive VR. These findings are consistent with the findings of Liu et al. [43], who conducted a meta-analysis that demonstrated substantial increases in GMFM-E (*p* < 0.001) in children with CP as a consequence of interventions with Nintendo Wii in comparison to conventional physical therapies. Similarly, Komariah et al. [44] reported significant improvements in GMFM in favor of non-immersive VR in a meta-analysis conducted in children with CP (*p* = 0.03), while immersive VR did not exhibit significant improvements (*p* = 0.24). Gross motor function enhancements may be attributed to task-specific repetition, sensorimotor stimulus, and the nudging of neuroplastic processes that are induced by VR-based interventions from a physiological perspective. Systems that operate in this manner are typically founded on voluntary movements that are directed toward a specific task. Through interactive activities, they not only facilitate activation but also facilitate motor planning, proprioceptive input, and postural adjustments [11,45]. Successful motor execution can be facilitated by visual–auditory feedback in VR games, which in turn facilitates motor learning by activating Hebbian plasticity or long-term potentiation in the cortical motor area of the brain [45]. Furthermore, the motivational component of non-immersive VR may have a beneficial impact on dopaminergic transmission, a known factor that influences attention and reward processing, as well as reinforcement learning, which is associated with the development of motor skills [46,47]. Nevertheless, it is imperative to underscore that dopaminergic modulation is merely one component of a neurochemical complex that is responsible for motor learning and necessitates the involvement of other neurotransmitters, including norepinephrine and acetylcholine [48]. Additionally, in certain instances, attentional dysregulation or maladaptive plasticity may result from dopaminergic overreaction; therefore, it is imperative to maintain a balance of stimulation in neurorehabilitation protocols [49,50].

### 4.2. Balance

Balance outcomes, as measured by the PBS, also showed significant improvements in favor of non-immersive VR (ES = 1.34). However, heterogeneity among studies was considerable (I^2^ = 93.42%). These findings align with Liu et al. [43], who reported in their meta-analysis and systematic review that children with CP achieved greater balance improvements after VR training compared with conventional physical therapy (*p* < 0.001). Similarly, Komariah et al. [44] found significant improvements in balance following non-immersive VR interventions in children with CP.

The positive effects of VR on balance may be explained by its ability to simultaneously engage vestibular, proprioceptive, and visual systems, which are central to postural control. VR tasks typically involve interactive activities that require dynamic weight shifting, balance responses to moving stimuli, and adaptations to varying visual feedback. These demands elicit both reactive and anticipatory postural adjustments [51]. Repetition of such tasks may further drive cerebellar adaptation and reinforce neuromuscular pathways that support automatic balance control [52].

Additionally, VR-based interventions enhance multisensory integration and attentional engagement, contributing to more efficient central processing of balance-related inputs. This, in turn, improves stability, reduces postural sway, and supports functional performance in daily activities [30].

### 4.3. Functional Independence

Significant improvements were also observed in functional independence (*p* < 0.001; ES = 0.99), although effect sizes were smaller compared with those for gross motor function and balance. This outcome is of high clinical relevance, as promoting independence in activities of daily living (ADLs) is a central goal in pediatric rehabilitation. The standardized mean difference close to one point indicates that children receiving non-immersive VR interventions achieved meaningful improvements in tasks such as dressing, grooming, bathing, and ambulating. These gains are associated with greater autonomy, reduced caregiver burden, and enhanced social participation [53].

Improvements in gross motor skills and postural control likely contributed to these functional outcomes by facilitating independence in everyday routines. However, functional independence depends not only on physical abilities but also on cognitive and behavioral factors—including motivation, attention, and decision-making [54]. Even modest increases in self-care or mobility capacity may yield long-term benefits, positively influencing self-esteem, school engagement, and quality of life [55].

Our findings are consistent with previous evidence. Tobaiqi et al. [56] reported significant gains in WeeFIM scores among children with CP following VR-assisted non-immersive virtual reality (VR) (*p* < 0.0001), while Komariah et al. [44] found that non-immersive VR interventions significantly improved daily living activities (WeeFIM, *p* < 0.006). Collectively, these results reinforce the potential of VR-based rehabilitation to foster independence, reduce reliance on caregivers, and promote inclusion in academic and social environments critical for long-term development.

### 4.4. Subgroup Analysis by Session Duration (Minutes)

Findings suggest that session length plays an important role in the effectiveness of non-immersive VR interventions. Sessions lasting longer than 45 min were associated with greater improvements in postural balance, likely due to increased exposure to task-specific stimuli and extended engagement time. Longer practice may also enhance neural plasticity by providing more opportunities for intensive motor learning and sustained attentional focus, both of which are essential in pediatric neurorehabilitation [57].

### 4.5. Subgroup Analysis by Training Duration

Interestingly, interventions of shorter duration (≤6 weeks) appeared to be more effective. This may be explained by heightened novelty, motivation, and adherence during the initial weeks of therapy. From a neuroplastic perspective, children may adapt more efficiently to new cortical configurations during a brief but intensive training period [58]. These results support the use of time-limited, targeted interventions that can elicit meaningful motor improvements in pediatric CP rehabilitation.

### 4.6. Subgroup Analysis by Total Sessions

Interventions comprising fewer than 18 sessions appeared more effective, possibly due to higher engagement and reduced cognitive or physical fatigue. Shorter programs may also be more feasible at lower intensities per session, avoiding overload in children. The observed benefits may reflect rapid neuromotor acquisition during early exposure to VR-based stimuli, consistent with motor learning principles, where initial phases are often associated with greater gains [47].

### 4.7. Limitations and Strengths

This review has some limitations. (i) Heterogeneity was high across studies (I^2^ > 85% for gross motor function and >93% for balance). (ii) Moderator analyses were based on a limited number of studies (only 3–4 per variable). (iii) Exercise intensity was inconsistently reported, with no standardized intensity scales applied in most trials. (iv) Several studies presented a high or moderate risk of bias. (v) The sample was restricted to children under 12 years with GMFCS levels I–III, limiting generalizability and excluding long-term follow-up outcomes. (vi) There was not enough data to perform subgroup analysis based on the degree of motor impairment (GMFCS I–III). Only a few studies disclosed the GMFCS classification, and most did not report results separately for each level. Future RCTs are advised to standardize the reporting of GMFCS levels to enable more precise subgroup comparisons. (vii), variability in intervention settings (clinical vs. home-based), therapist supervision, and differences in non-immersive VR platforms may have contributed to heterogeneity.

Despite these limitations, the study presents notable strengths. It adhered rigorously to PRISMA guidelines and was prospectively registered in PROSPERO. The search strategy was comprehensive, covering six major databases (PubMed, Web of Science, Scopus, MEDLINE, CINAHL Complete, and Psychology and Behavioral Sciences Collection) without language restrictions. Methodological quality was systematically assessed using TESTEX, while RoB 2 and GRADE were applied to evaluate risk of bias and certainty of evidence. Robust statistical approaches were employed, including Hedges’ g, random-effects modeling, heterogeneity assessment, and moderator/subgroup analysis. Finally, the review focused on clinically relevant outcomes (GMFM-D, GMFM-E, PBS, and WeeFIM), ensuring practical applicability to pediatric rehabilitation.

### 4.8. Practical Applications

Based on the findings of this meta-analysis, non-immersive VR systems such as the Nintendo Wii and Xbox Kinect are recommended to enhance gross motor function and balance in children with CP. Optimal results were observed with sessions lasting more than 45 min, delivered 2–5 times per week, for a total of fewer than 18 sessions across a maximum of 6 weeks. These parameters appear most effective in improving GMFCS levels and balance outcomes.

Progress should be routinely monitored using GMFM-D/E, PBS, and WeeFIM at baseline and post-intervention. Training intensity should be adapted to individual tolerance, while adherence and any adverse effects are recorded to guide workload adjustments. In home-based programs, caregiver training and, when possible, telemonitoring are recommended to ensure safe and consistent practice. In clinical or school-based environments, direct supervision allows for real-time adaptations. Finally, combining non-immersive VR with conventional therapies or incorporating varied tasks in extended programs may help sustain motivation and adherence without increasing risk [59,60].

### 4.9. Epidemiological Applications

Non-immersive VR also holds promise for broader epidemiological applications, particularly in underserved populations. As a relatively low-cost and widely accessible technology, it can be integrated into community and school-based rehabilitation programs [60]. Incorporating VR into health strategies may also support early intervention policies, enabling more equitable neurodevelopmental outcomes in children with CP [61]. From a public health perspective, coupling non-immersive VR with tele-rehabilitation could help address service delivery gaps and provide continuous monitoring of functional outcomes at the population level [56].

## 5. Conclusions

This meta-analysis provides strong evidence that non-immersive VR can enhance gross motor function, balance, and functional independence in children with CP. Significant improvements were observed in GMFM-D, GMFM-E, PBS, and WeeFIM scores in favor of non-immersive VR compared with control conditions. These findings highlight the value of commercially available VR gaming systems such as the Xbox Kinect and Nintendo Wii as engaging, motivating, and accessible tools for pediatric neurorehabilitation.

Subgroup analysis suggests that interventions lasting at least 45 min per session and totaling fewer than 18 sessions yield the most favorable outcomes. Overall, non-immersive VR represents a feasible and promising approach for integration into both clinical and home-based physical therapy programs for children with CP.

## Figures and Tables

**Table 1:** Selection criteria used in the systematic review.

Category	Inclusion Criteria	Exclusion Criteria
Population	Participants under 12 years of age, diagnosed with cerebral palsy, regardless of sex [22].	Single clinical case reports or studies including adults (>18 years).
Intervention	Interventions using non-immersive virtual reality or active non-immersive video games (e.g., Wii Sports, Kinect, Switch Sports), with a minimum duration of 2 weeks.	Immersive or semi-immersive VR; interventions lacking procedural detail.
Comparator	Active controls (e.g., conventional physiotherapy, task-specific, or neurodevelopmental training) or inactive controls (usual care or daily activities).	Studies without control groups.
Outcome	At least one pre- and post-intervention assessment of gross motor function (GMFM-D/E), balance (PBS), or functional independence (WeeFIM).	Studies not reporting baseline or follow-up outcomes.
Study Design	Randomized controlled trials with pre- and post-assessments.	Nonrandomized studies, protocols, or reviews.

GMFM-D/E: Gross motor function measure, Dimensions D (Standing) and E (Walking, Running, and Jumping); PBS: Pediatric Balance Scale; VR: Virtual Reality; WeeFIM: Functional Independence Measure for Children.

**Table 2:** Study quality assessment according to the TESTEX scale.

Study	Eligibility Criteria Specified	Randomly Allocated Participants	Allocation Concealed	Groups Similar at Baseline	Assessors Blinded	Outcome Measures Assessed > 85% of Participants *	Intention to Treat Analysis	Reporting of Between-Group Statistical Comparisons	Point Measures and Measures of Variability Reported **	Activity Monitoring in the Control Group	Relative Exercise Intensity Reviewed	Exercise Volume and Energy Expended	Overall TESTEX #
[9]	Yes	Yes	No	Yes	No	Yes (3)	No	Yes	Yes (2)	No	No	No	8/15
[17]	Yes	Yes	Yes	Yes	No	Yes (1)	No	Yes	Yes (2)	No	No	No	8/15
[18]	Yes	Yes	No	Yes	No	Yes (1)	No	Yes	Yes (2)	No	No	No	9/15
[19]	Yes	Yes	Yes	Yes	Yes	Yes (1)	No	Yes	Yes (2)	No	Yes	No	10/15
[34]	Yes	Yes	Yes	Yes	Yes	Yes (2)	Yes	Yes	Yes (2)	No	Yes	No	12/15
[35]	Yes	Yes	No	Yes	Yes	Yes (1)	No	Yes	Yes (2)	No	Yes	No	9/15
[36]	Yes	Yes	Yes	Yes	Yes	Yes (1)	No	Yes	Yes (2)	No	No	No	9/15
[37]	Yes	Yes	Yes	Yes	Yes	No	No	Yes	Yes (2)	Yes	No	No	9/15
[38]	Yes	Yes	Yes	Yes	No	Yes (1)	No	Yes	Yes (2)	No	Yes	No	9/15
[39]	Yes	Yes	Yes	Yes	No	Yes (1)	No	Yes	Yes (2)	No	Yes	No	9/15
[40]	Yes	Yes	No	Yes	Yes	Yes (1)	No	Yes	Yes (2)	No	No	No	9/15
[41]	Yes	Yes	Yes	Yes	Yes	Yes (1)	No	Yes	Yes (2)	No	Yes	No	10/15
[42]	Yes	Yes	Yes	Yes	Yes	Yes (2)	No	Yes	Yes (2)	No	No	No	11/15

* Three points are possible: one point if adherence >85%, one point if adverse events were reported, and one point if exercise attendance was reported. ** Two points possible: one point if the primary outcome is reported, and one point if all other outcomes are reported. # Total out of 15 points. TESTEX: Tool for assessing study quality and reporting in exercise.

**Table 3:** Characteristics of participants examined in the included studies.

Author	Country	CP Type	Groups (n)	Mean Age (y)	Console/Device	Settings	Training Volume			Outcomes Evaluated	Main Results
							Week	Frequency (Sessions/Weeks)	Session Duration (Min)		
[9]	IN	NR	VRT: 8; CG: 8	VRT: 8.8 ± 3.2; CG: 10.3 ± 4.4	Nintendo Wii Fit/Wii Fit Plus	NR	3	3	30	PBS, MACS	↑ PBS, MACS ↔
[17]	TR	Spastic	VRT: 27; CG: 25	VRT: 9.2 ± 2.0; CG: 9.4 ± 2.2	Xbox Kinect	Clinic	8	2	45	PBS, GMFM-88	↑ PBS, GMFM-88 ↔
[18]	TR	Dysplegic, hemiplegic, dyskinetic	VRT: 15; CG: 15	VRT: 10.2.6; CG: 10.5 ± 2.7	Nintendo Wii/Xbox Kinect	Clinic	12	2	50	WeeFIM	↑ WeeFIM
[19]	EG	Ataxic	VRT: 32; CG: 32	VRT: 10.7 ± 1.2; CG: 11.2 ± 1.4	Nintendo Wii/Xbox Kinect	Clinic	12	3	60	PBS	↑ PBS
[34]	IN	Bilateral spastic	VRT: 19; CG: 19	VRT: 8.9 ± 1.9; CG: 8.7 ± 1.6	Customized Kinect-based VR system	Clinic	6	4	60	GMFM-88, PBS, WeeFIM	GMFM-88 ↔, ↑ PBS, WeeFIM ↔
[35]	SK	Spastic	VRT: 9; CG: 9	VRT: 10.2 ± 3.4; CG: 9.4 ± 3.8	Treadmill with VR	Clinic	8	3	30	GMFM-E, PBS	↑ GMFM-E, ↑ PBS
[36]	BR	NR	VRT: 10; CG: 10	VRT: 7.6 ± 2.2; CG: 7.4 ± 2.0	Not specified	Clinic	2	5	20	PBS, TUG	↑ PBS, ↑ TUG
[37]	UK	NR	VRT: 8; CG: 12	VRT: 9.7 ± 2.1; CG: 9.5 ± 2.3	Nintendo Wii/Wii Fit	Home	12	3	30	GMFM-66, TUG	GMFM-66 ↔, TUG ↔
[38]	TW	Spastic	VRT: 13; CG: 14	VRT: 8.7 ± 2.1; CG: 8.6 ± 2.2	Electro Sync Cycle VR system	Home	12	3	40	GMFM-66	↑ GMFM-66
[39]	CN	NR	VRT1: 15; VRT2: 15; VRT3: 15; CG: 15	VRT1: 5.0 ± 1.6; VRT2: 5.3 ± 2.9; VRT3: 5.6 ± 1.5; CG: 4.5 ± 1.7	Nintendo Wii	Clinic	12	4	50	GMFM-D, GMFM-E	↑ GMFM-D, ↑ GMFM-E
[40]	TR	Unilateral spastic	VRT: 30; CG: 30	VRT: 10.5 ± 3.6; CG: 10.0 ± 3.2	Nintendo Wii/Xbox Kinect	Clinic	8	2	45	NR	NR
[41]	TW	Unilateral	VRT: 9; CG: 9	VRT: 8.5 ± 2.0; CG: 8.5 ± 2.1	Custom VR device	Home	8	2	135	WeeFIM	↑ WeeFIM
[42]	LB	Spastic hemiplegia, diplegia, monoplegia	VRT: 14; CG: 15	VRT: 8.2 ± 2.0; CG: 7.4 ± 3.0	Custom VR systems	Clinic	6	3	60	PBS, GMFM-D, GMFM-E	PBS ↔, GMFM-D ↔, GMFM-E ↔

BR: Brazil; CG: Control Group; CN: China; CP: Cerebral Palsy; EG: Egypt; GMFM-66: Gross motor function Measure–66; GMFM-88: Gross motor function Measure–88; GMFM-D: Gross motor function Measure, Dimension D (Standing); GMFM-E: Gross motor function Measure, Dimension E (Walking, Running, Jumping); IN: India; LB: Lebanon; MACS: Manual Ability Classification System; NR: Not Reported; PBS: Pediatric Balance Scale; SK: South Korea; tDCS: Transcranial Direct Current Stimulation; ↑ improvement compared with the control group (CG); ↓ worsening compared with CG; ↔ no change between groups. TR: Turkey; TUG: Timed Up and Go; TW: Taiwan; UK: United Kingdom; VRT: Virtual Reality Training; WeeFIM: Functional Independence Measure for Children.

**Table 4:** Summary of findings from the studies included the effects of non-immersive VR on the GMF, balance, and functional independence in children with CP.

Gross Motor Function, Balance, and Functional Independence	n of Studies	n of Experimental Groups	n of Control Groups	Total Participants	ES (95%CI)	*p*	I^2^ (%)	Egger’s Test (*p*)	RW (%)
Gross motor function									
GMFM-D	5	5	5	137	2.04 (0.73 to 3.35)	**0.02**	89.86	<0.001	0.14 to 0.22
GMFM-E	5	5	5	137	2.04 (0.97 to 3.08)	**<0.001**	84.70	<0.001	0.34 to 0.52
Balance									
PBS	7	7	7	237	1.34 (0.17 to 2.52)	**0.02**	93.42	<0.001	0.13 to 0.17
Functional Independence									
WeeFIM	4	4	4	146	0.99 (0.65 to 1.33)	**<0.001**	32.15	0.21	3.95 to 6.22

Bolded *p*-values indicate significant improvement (*p* < 0.05) in the experimental group after the non-immersive VR intervention compared with the control group. CI: Confidence Interval; ES: Effect Size (Hedges’ g); GMFM-D: Gross Motor Function Measure, Dimension D (Standing); GMFM-E: Gross Motor Function Measure, Dimension E (Walking, Running, Jumping); I^2^: Heterogeneity Index; PBS: Pediatric Balance Scale; RW: Relative Weight; WeeFIM: Functional Independence Measure for Children.

**Table 5:** Grading of Recommendations, Assessment, Development, and Evaluation (GRADE) scale.

Certainty of Evidence							Nº of Patients		Effect		Certainty	Importance
Nº of Studies	Study Design	Risk Assessment	Inconsistency	Indirect Evidence	Vagueness	Other Considerations	[Intervention]	[Comparison]	Relative (95% CI)	Absolute (95% CI)		
Gross motor function												
4	RCT	It is not serious	It is not serious	It is not serious	It is not serious	None	69/137 (50.4%)	68/137 (49.6%)	Not estimable		++++ High	IMPORTANT
Balance												
7	RCT	Serious ^a^	It is not serious	It is not serious	It is not serious	None	119/237 (50.2%)	118/237 (49.8%)	Not estimable		+++ Moderate	IMPORTANT
Functional Independence												
4	RCT	Serious ^a^	It is not serious	It is not serious	It is not serious	None	73/146 (50.0%)	73/146 (50.0%)	Not estimable		+++ Moderate	IMPORTANT

CI: Confidence Interval; ^a^ Some concerns.

## Data Availability

The datasets generated during and/or analyzed during the current research are available from the corresponding author upon reasonable request.

## Supplementary Materials

The following supporting information can be downloaded at https://www.mdpi.com/article/10.3390/jcm14217582/s1. Checklist S1. PRISMA 2020 Checklist. Figure S1. Forest plot of changes in GMFM-D dimension scores in children with cerebral palsy following Virtual reality training interventions compared with a control group. Values shown correspond to effect sizes (Hedges’ g) with 95% confidence intervals (CI). The squares represent the effect sizes of each study, while the size of each square reflects the statistical weight of each study within the meta-analysis. Positive values favor children receiving the exergames intervention, while negative values favor the control group; Figure S2. Forest plot of changes in GMFM-E dimension scores in children with cerebral palsy following Virtual reality training interventions compared with a control group. Values shown correspond to effect sizes (Hedges’ g) with 95% confidence intervals (CI). The squares represent the effect sizes of each study, while the size of each square reflects the statistical weight of each study within the meta-analysis. Positive values favor children receiving the exergames intervention, while negative values favor the control group; Figure S3. Forest plot of changes in Pediatric Balance Scale (PBS) scores in children with cerebral palsy following Virtual reality training interventions compared with a control group. Values shown correspond to effect sizes (Hedges’ g) with 95% confidence intervals (CI). The squares represent the effect sizes of each study, while the size of each square reflects the statistical weight of each study within the meta-analysis. Positive values favor children receiving the exergames intervention, while negative values favor the control group; Figure S4. Forest plot of changes in functional independence (WeeFIM scores) in children with cerebral palsy following Virtual reality training interventions compared with a control group. Values shown correspond to effect sizes (Hedges’ g) with 95% confidence intervals (CI). The squares represent the effect sizes of each study, while the size of each square reflects the statistical weight of each study within the meta-analysis. Positive values favor children receiving the exergames intervention, while negative values favor the control group; Figure S5. Forest plot of changes in performance on the Pediatric Balance Scale (PBS) in children with cerebral palsy following Virtual reality training interventions compared to a control group, stratified by session duration. The values shown correspond to effect sizes (Hedges’ g) with 95% confidence intervals (CI). The squares represent the effect sizes of each study, with their size indicating the statistical weight of each study within the meta-analysis. The studies are divided into two subgroups: sessions lasting 45 min or less and sessions lasting more than 45 min. Positive values favor children in the exergames intervention, while negative values favor the control group. The red diamonds represent the pooled effect sizes for each subgroup (Figure S6). Forest plot of changes in performance on the Pediatric Balance Scale (PBS) in children with cerebral palsy following Virtual reality training interventions compared to a control group, stratified by intervention duration. The values shown correspond to effect sizes (Hedges’ g) with 95% confidence intervals (CI). The squares represent the effect sizes of each study, with their size indicating the statistical weight of each study within the meta-analysis. The studies are divided into two subgroups: interventions lasting 6 weeks or less and interventions lasting more than 6 weeks. Positive values favor children in the exergames intervention, while negative values favor the control group. The red diamonds represent the pooled effect sizes for each subgroup (Figure S7). Forest plot of changes in performance on the Pediatric Balance Scale (PBS) in children with cerebral palsy following Virtual reality training interventions compared to a control group, stratified by total number of sessions. The values shown correspond to effect sizes (Hedges’ g) with 95% confidence intervals (CI). The squares represent the effect sizes of each study, with their size indicating the statistical weight of each study within the meta-analysis. The studies are divided into two subgroups: interventions with 18 sessions or fewer and interventions with more than 18 sessions. Positive values favor children in the exergames intervention, while negative values favor the control group. The red diamonds represent the pooled effect sizes for each subgroup.
