# Supplementary material for: Effectiveness of Non-Immersive Virtual Reality on Gross Motor Function, Balance, and Functional Independence in Children with Cerebral Palsy: A Systematic Review with Meta-Analysis
Source: J Clin Med. 2025 Oct 25;14(21):7582. doi: 10.3390/jcm14217582 (PMC12608441; doi:10.3390/jcm14217582)
Supplement: Supplementary file 1 [file jcm-14-07582-s001.zip › jcm-3904966-supplementary.pdf]

Supplementary Materials

Study name	Hedges's g	Standard error	Variance	Lower limit	Upper limit	Z-Value	p-Value
	Statistics for each study						
Cho et al. (2016)	0,092	0,449	0,202	-0,788	0,973	0,205	0,837
Fu et al. (2022) A	1,152	0,385	0,148	0,397	1,907	2,990	0,003
Fu et al. (2022) B	5,608	0,806	0,650	4,027	7,189	6,954	0,000
Fu et al. (2022) C	2,249	0,459	0,210	1,349	3,148	4,901	0,000
Ziab et al. (2024)	1,761	0,429	0,184	0,920	2,602	4,106	0,000
	2,045	0,667	0,445	0,738	3,351	3,066	0,002

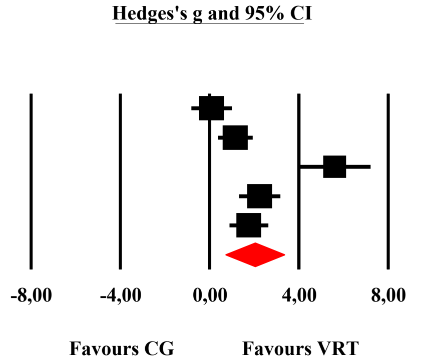

Figure S1. Forest plot of changes in GMFM-D dimension scores in children with cerebral palsy following Virtual reality training interventions compared with a control group. Values shown correspond to effect sizes (Hedges' g) with 95% confidence intervals (CI). The squares represent the effect sizes of each study, while the size of each square reflects the statistical weight of each study within the meta-analysis. Positive values favor children receiving the exergames intervention, while negative values favor the control group.

Study name	Hedges's g	Standard error	Variance	Lower limit	Upper limit	Z-Value	p-Value
	Statistics for each study						
Cho et al. (2016)	0,508	0,457	0,209	-0,387	1,404	1,112	0,266
Fu et al. (2022) A	1,881	0,430	0,185	1,037	2,724	4,371	0,000
Fu et al. (2022) B	4,702	0,703	0,495	3,323	6,080	6,685	0,000
Fu et al. (2022) C	2,074	0,445	0,198	1,202	2,946	4,662	0,000
Ziab et al. (2024)	1,449	0,408	0,167	0,649	2,249	3,550	0,000
	2,029	0,540	0,292	0,971	3,088	3,758	0,000

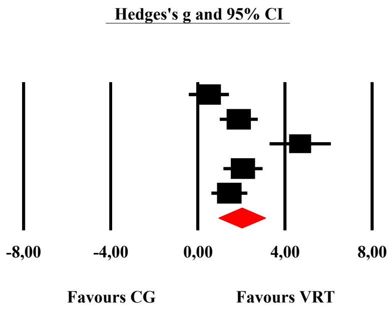

Figure S2. Forest plot of changes in GMFM-E dimension scores in children with cerebral palsy following Virtual reality training interventions compared with a control group. Values shown correspond to effect sizes (Hedges' g) with 95% confidence intervals (CI). The squares represent the effect sizes of each study, while the size of each square reflects the statistical weight of each study within the meta-analysis. Positive values favor children receiving the exergames intervention, while negative values favor the control group.

Study name	Hedges's g	Standard error	Variance	Lower limit	Upper limit	Z-Value	p-Value
Cho et al. (2016)	0,175	0,450	0,202	-0,707	1,057	0,389	0,698
Jha et al. (2021)	0,584	0,325	0,105	-0,053	1,220	1,798	0,072
Sharan et al. (2012)	0,025	0,473	0,223	-0,901	0,952	0,054	0,957
Lazzari et al. (2017)	0,453	0,434	0,189	-0,398	1,304	1,043	0,297
Fidan et al. (2023)	0,247	0,274	0,075	-0,291	0,785	0,900	0,368
Mouhamed et al. (2024)	4,072	0,437	0,191	3,217	4,928	9,329	0,000
Ziab et al. (2024)	4,173	0,656	0,431	2,887	5,460	6,359	0,000
	1,346	0,599	0,359	0,172	2,521	2,247	0,025

Statistics for each study

Hedges's g and 95% CI

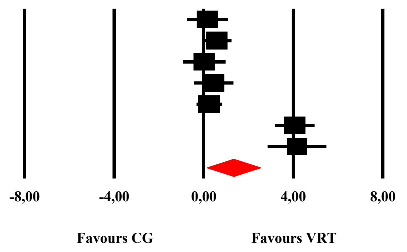

Figure S3. Forest plot of changes in Pediatric Balance Scale (PBS) scores in children with cerebral palsy following Virtual reality training interventions compared with a control group. Values shown correspond to effect sizes (Hedges' g) with 95% confidence intervals (CI). The squares represent the effect sizes of each study, while the size of each square reflects the statistical weight of each study within the meta-analysis. Positive values favor children receiving the exergames intervention, while negative values favor the control group.

Study name	Hedges's g	Standard error	Variance	Lower limit	Upper limit	Z-Value	p-Value
Jha et al. (2021)	0,912	0,334	0,112	0,256	1,567	2,727	0,006
Sahin et al. (2020)	1,104	0,274	0,075	0,567	1,641	4,029	0,000
Tarakci et al. (2016)	0,516	0,361	0,131	-0,192	1,225	1,428	0,153
Wang et al. (2021)	1,846	0,544	0,296	0,780	2,913	3,392	0,001
	1,005	0,217	0,047	0,579	1,430	4,625	0,000

Statistics for each study

Hedges's g and 95% CI

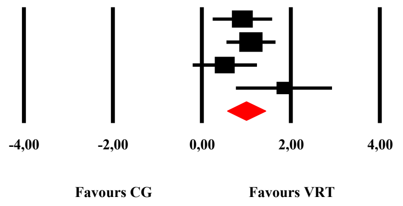

Figure S4. Forest plot of changes in functional independence (WeeFIM scores) in children with cerebral palsy following Virtual reality training interventions compared with a control group. Values shown correspond to effect sizes (Hedges' g) with 95% confidence intervals (CI). The squares represent the effect sizes of each study, while the size of each square reflects the statistical weight of each study within the meta-analysis. Positive values favor children receiving the exergames intervention, while negative values favor the control group.

Group by Subgroup within study	Study name	Subgroup within study	Hedges's g	Standard error	Variance	Lower limit	Upper limit	Z-Value	p-Value
<45 minutes	Cho et al. (2016)	<45 minutes	0,175	0,450	0,202	-0,707	1,057	0,389	0,698
<45 minutes	Sharan et al. (2012)	<45 minutes	0,025	0,473	0,223	-0,901	0,952	0,054	0,957
<45 minutes	Lazzari et al. (2017)	<45 minutes	0,453	0,434	0,189	-0,398	1,304	1,043	0,297
<45 minutes			0,230	0,261	0,068	-0,281	0,740	0,881	0,378
>45 minutes	Jha et al. (2021)	>45 minutes	0,584	0,325	0,105	-0,053	1,220	1,798	0,072
>45 minutes	Fidan et al. (2023)	>45 minutes	0,247	0,274	0,075	-0,291	0,785	0,900	0,368
>45 minutes	Mouhamed et al. (2024)	>45 minutes	4,072	0,437	0,191	3,217	4,928	9,329	0,000
>45 minutes	Ziab et al. (2024)	>45 minutes	4,173	0,656	0,431	2,887	5,460	6,359	0,000
>45 minutes			2,217	0,989	0,978	0,278	4,155	2,241	0,025

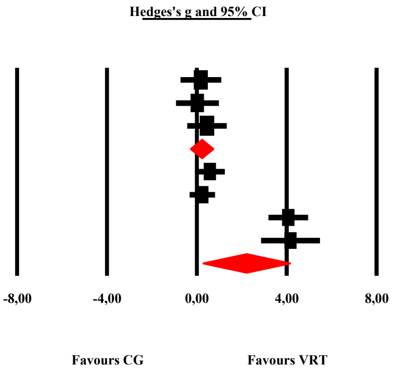

Figure S5. Forest plot of changes in performance on the Pediatric Balance Scale (PBS) in children with cerebral palsy following Virtual reality training interventions compared to a control group, stratified by session duration. The values shown correspond to effect sizes (Hedges' g) with 95% confidence intervals (CI). The squares represent the effect sizes of each study, with their size indicating the statistical weight of each study within the meta-analysis. The studies are divided into two subgroups: sessions lasting 45 minutes or less and sessions lasting more than 45 minutes. Positive values favor children in the exergames intervention, while negative values favor the control group. The red diamonds represent the pooled effect sizes for each subgroup.

Group by Subgroup within study	Study name	Subgroup within study	Hedges's g	Standard error	Variance	Lower limit	Upper limit	Z-Value	p-Value
<6 weeks	Jha et al. (2021)	<6 weeks	0,584	0,325	0,105	-0,053	1,220	1,798	0,072
<6 weeks	Sharan et al. (2012)	<6 weeks	0,025	0,473	0,223	-0,901	0,952	0,054	0,957
<6 weeks	Lazzari et al. (2017)	<6 weeks	0,453	0,434	0,189	-0,398	1,304	1,043	0,297
<6 weeks	Ziab et al. (2024)	<6 weeks	4,173	0,656	0,431	2,887	5,460	6,359	0,000
<6 weeks			1,228	0,716	0,512	-0,175	2,631	1,715	0,086
>6 weeks	Cho et al. (2016)	>6 weeks	0,175	0,450	0,202	-0,707	1,057	0,389	0,698
>6 weeks	Fidan et al. (2023)	>6 weeks	0,247	0,274	0,075	-0,291	0,785	0,900	0,368
>6 weeks	Mouhamed et al. (2024)	>6 weeks	4,072	0,437	0,191	3,217	4,928	9,329	0,000
>6 weeks			1,488	1,210	1,465	-0,884	3,861	1,230	0,219

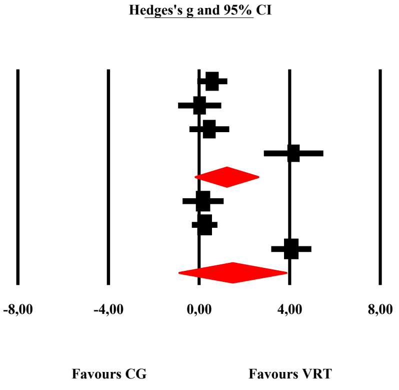

Figure S6. Forest plot of changes in performance on the Pediatric Balance Scale (PBS) in children with cerebral palsy following Virtual reality training interventions compared to a control group, stratified by intervention duration. The values shown correspond to effect sizes (Hedges' g) with 95% confidence intervals (CI). The squares represent the effect sizes of each study, with their size indicating the statistical weight of each study within the meta-analysis. The studies are divided into two subgroups: interventions lasting 6 weeks or less and interventions lasting more than 6 weeks. Positive values favor children in the exergames intervention, while negative values favor the control group. The red diamonds represent the pooled effect sizes for each subgroup.

Group by Subgroup within study	Study name	Subgroup within study	Hedges's g	Standard error	Variance	Lower limit	Upper limit	Z-Value	p-Value
			Statistics for each study						
< 18 total sessions	Sharan et al. (2012)	< 18 total sessions	0,025	0,473	0,223	-0,901	0,952	0,054	0,957
< 18 total sessions	Lazzari et al. (2017)	< 18 total sessions	0,453	0,434	0,189	-0,398	1,304	1,043	0,297
< 18 total sessions	Fidan et al. (2023)	< 18 total sessions	0,247	0,274	0,075	-0,291	0,785	0,900	0,368
< 18 total sessions	Ziab et al. (2024)	< 18 total sessions	4,173	0,656	0,431	2,887	5,460	6,359	0,000
< 18 total sessions			1,135	0,716	0,513	-0,269	2,539	1,584	0,113
> 18 total sessions	Cho et al. (2016)	> 18 total sessions	0,175	0,450	0,202	-0,707	1,057	0,389	0,698
> 18 total sessions	Jha et al. (2021)	> 18 total sessions	0,584	0,325	0,105	-0,053	1,220	1,798	0,072
> 18 total sessions	Mouhamed et al. (2024)	> 18 total sessions	4,072	0,437	0,191	3,217	4,928	9,329	0,000
> 18 total sessions			1,605	1,171	1,372	-0,691	3,900	1,370	0,171

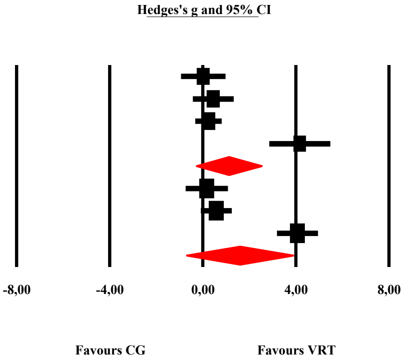

Figure S7. Forest plot of changes in performance on the Pediatric Balance Scale (PBS) in children with cerebral palsy following Virtual reality training interventions compared to a control group, stratified by total number of sessions. The values shown correspond to effect sizes (Hedges' g) with 95% confidence intervals (CI). The squares represent the effect sizes of each study, with their size indicating the statistical weight of each study within the meta-analysis. The studies are divided into two subgroups: interventions with 18 sessions or fewer and interventions with more than 18 sessions. Positive values favor children in the exergames intervention, while negative values favor the control group. The red diamonds represent the pooled effect sizes for each subgroup.
